# Commentary: Phage Therapy of Staphylococcal Chronic Osteomyelitis in Experimental Animal Model

**DOI:** 10.3389/fmicb.2016.01251

**Published:** 2016-08-10

**Authors:** Stephen T. Abedon

**Affiliations:** Department of Microbiology, The Ohio State UniversityMansfield, Ohio, USA

**Keywords:** phage therapy, chronic infection, antibacterial agents, bacteriophages, staphylococcal infections, animal models

Phage therapy—the use of bacterial viruses as equivalents of antibacterial “drugs”—has been practiced for nearly 100 years. Much, particularly since the 1940s, has taken place in the former Soviet Union. Nevertheless, interest in phage therapy in Western countries has been steadily building since the 1980s. Much of this interest has been in response to growing concerns over antibiotic resistance, though there are concerns, as well, over antibiotic side effects (Langdon et al., 2016). Phage therapy in many cases has been used as a means of treating chronic bacterial infections which may be *tolerant* to antibiotic treatment rather than necessarily also genetically resistant. This antibiotic tolerance is thought to occur predominantly as a consequence of bacterial growth within biofilms (Olsen, 2015). In practice, however, what is going on is that phage therapy has been used to treat bacterial infections against which antibiotics, often following months or years of treatment, have not been successful (e.g., Rhoads et al., 2009; Wright et al., 2009; Miedzybrodzki et al., 2012).

Despite this clinical aspect of phage therapy, pre-clinical studies have not tended to focus experimentally on chronic bacterial infections. There have been exceptions, however, particularly in which a fair amount of time has elapsed between bacterial challenge and start of phage treatment (Table 1). Here I focus on the recent study of Kishor et al. (2016), which employs the longest delay of which I am aware, in an animal model, between bacterial challenge and phage application.

**Table 1:** **Animal models of human phage therapy with treatment delays exceeding 24 h^a^**.

**Organism(s) treated**	**Delay before treatment^b^**	**Challenge**	**Consequence**	**Treatment**	**Results^c^**	**References**
*Escherichia coli*	168 h	Force-fed axenic mice with 5 × 10^7^ CFU	Intestinal colonization	10^5^ PFU/ml in drinking water	~10^8^ CFU/ml reduced to ~10^4^ CFU/ml in feces	Chibani-Chennoufi et al., 2004
*Escherichia coli* O157:H7	48 h	Force-fed mice with 10^9^ CFU	Intestinal colonization	Force fed 10^8^ PFU, 1 dose, or 10^10^ PFU, 1 dose or “daily”	< 10^3^ vs. < 10^2^ CFU/g in feces for control (etc.) vs. “daily” treatment, around day 8	Tanji et al., 2005
*Mycobacterium avium*	168 h	IV 3 × 10^7^ CFU to mice	Continuing replication	IV 8 × 10^9^ PFU or 4 × 10^7^ phage-infected *M. smegmatis* in 100 μl, 1 or 2 doses	~0.5-log fewer CFU in spleen for phage-infected *M. smegmatis*, day 14, no reduction for phage only	Danelishvili et al., 2006
*Staphylococcus aureus*	96 h	Subcutaneous 10^8^ or 10^9^ CFU/mouse	Abscess development	Subcutaneous 10^9^ PFU^d^ in 200 μl, 1 or 4 doses	~10^8^ vs. ~10^6^ or ~10^4^ CFU per abscess for control vs. treatments	Capparelli et al., 2007
*Staphylococcus aureus*	240 h	IV 5 × 10^6^ CFU to mice	Non-lethal systemic infection	10^9^ PFU in 200 μl^e^	~10^4^ CFU vs. 0 in various organs, day 20	Capparelli et al., 2007
*Pseudomonas aeruginosa*	96, 240 h^f^	10^8^ CFU/ml in mouse drinking water^g^	Gut-derived septicemia	10^10^ PFU in 100 μl orally administered	0, 10, and 66.7% 20-day survival for no, 240, and 96-h treatments	Watanabe et al., 2007
*Salmonella enterica*	48, 336 h	IV 10^6^ or 10^5^ CFU, respectively, to mice	Sublethal systemic infection	IV 10^7^ PFU in 100 μl	~10^4^ CFU reduced to 0 in various organs	Capparelli et al., 2010
*Acinetobacter baumannii, Pseudomonas aeruginosa, Staphylococcus aureus*	96 h	Mouse wounds injected with 100 μl of organisms of one type	Infected wound (diabetes mellitus model)	10^8^–10^9^ PFU presumably topical following one or more debridements, >10 doses	~10^5^ vs. ~10^2^ CFU/swab, day 8, control vs. treatment (*S. aureus*)^h^	Mendes et al., 2013
*Mycobacterium ulcerans*	792 h	Subcutaneous 3 × 10^5^ CFU into mouse footpad	Footpad swelling with continuing replication	Subcutaneous 10^8^ PFU into mouse footpad	~3 × 10^5^ vs. ~3 × 10^3^ CFU/footpad, day 68, control vs. treatment	Trigo et al., 2013
*Staphylococcus aureus* (MRSA)	96 h	Intranasal 10^6^ CFU/mouse	Nare potentially transient colonization	Intranasal 50 μl of 10^7^ PFU/ml, 2 doses	~10^5^.5 vs. ~10^1^ CFU/g, day 11, control vs. treatment	Chhibber et al., 2014
*Staphylococcus aureus*	168 h	Sinus inoculation of sheep	Sinusitis model	Sinus inoculation of 100 ml of 2 × 10^8^ PFU/ml, 5 doses	~80% reduction in biofilm biomass, control vs. treatment	Drilling et al., 2014
*Acinetobacter baumannii* (multi-drug resistant)	48 h	10^8^ CFU inoculated into wound of diabetic rats	Abscess development	400 μl of 3 × 10^9^/ml PFU sprayed onto debrided wound	~10^9^ vs. 0 CFU, day 8, control vs. treatment	Shivaswamy et al., 2015
*Klebsiella pneumoniae*	48, 72 h	Intranasal 10^4^ CFU/mouse	Lobar pneumonia	IP, liposome-entrapped phages^i^	~10^5^ vs. ~10^3^ vs. 0 CFU, day 5, control vs. 72-h delay vs. 48-h delay^j^	Singla et al., 2015
*Staphylococcus aureus* (MRSA)	504, 1008 h^k^	Intramedullary injection of rabbits with uncertain number (≤ 5 × 10^6^) CFU	Chronic osteomyelitis	Intralesional injection of 5 × 10^11^ PFU, 4 doses	Cure of infection versus (for 504 h delay) lack of cure w/o treatment	Kishor et al., 2016

a *CFU, Colony-Forming Units; IP, Intraperitoneal; IV, Intravenous; MRSA, Methicillin-Resistant or Multi-drug Resistant Staphylococcus aureus; PFU, Plaque-Forming Units*.

b *Time between initial exposure to bacteria and initial exposure to phages. Shown per study are only those delays of longer than 24 h*.

c *Day indicated is post initial bacterial challenge*.

d *It is difficult to tell whether the phage administration was or was not made directly to the abscess*.

e *Phages were presumably delivered intravenously, though this is not explicitly stated*.

f *Phage additions were either 48- to 96-h prior to (96-h treatment) or 48- to 96-h post (240-h treatment) the cyclophosphamide-mediated induction of septicemia*.

g *This was followed days later by cyclophosphamide IP injection to induce septicemia*.

h *P. aeruginosa saw similar drops without phage treatment though sooner with phage treatment; A. baumanni also saw substantial drops with phage treatment when assaying for colony counts using selective media; mostly analogous though not identical and also more variable results were seen for S. aureus and P. aeruginosa with an otherwise equivalent pig model*.

i *Both free and liposome-entrapped phages were administered in this study representing concentrations of 10^9^ and 10^7^ PFU/ml and multiplicities of infection of 1 and 0.01, respectively*.

j *No difference in results was observed between control and 48-h delay in phage treatment w/o liposome entrapment. Note that 5-days post bacterial challenge is 3 and 2 days post 48 and 72-h phage treatment, respectively. The 72-h treatment also was reduced to zero CFU 7 days post bacterial challenge while the no-treatment control was reduced to 10^3^ CFU/ml at 7 days*.

k *3 and 6 weeks, respectively, though the 3-week treatment may in fact have actually begun on day 16*.

Kishor et al. (2016) present a rabbit model of chronic osteomyelitis caused by a methicillin-resistant isolate of *Staphylococcus aureus*. Two rabbits were used to test phage cocktail safety (intraperitoneal delivery of ~10^11^ plaque-forming units), four served as untreated controls, 12 were treated with phages after ~3 weeks, and another four were treated with phages after 6 weeks. Cocktails consisted of seven phages and a total of four doses were applied, 2 days between each. Wounds were prepared thusly (p. 89): “…a 2 cm long incision was made at the lateral aspect of distal end of femur and metaphysial area was exposed. With the help of a hand drill, a 5 mm diameter unicortical defect was created…” Following bacterial inoculation, “The incised area was covered by sterile bandages with one stitch at middle of open area.” Phage treatment by injection into the infected area, with either 3- and 6-week delays, resulted in negative cultures in 15 cases. The 16^th^ was sacrificed for study prior to completion of phage administration.

I have a few minor criticisms of the study, which the authors note is “preliminary.” First, it is difficult to tell just how many bacteria were applied. Second, all control rabbits were sacrificed during week 6, meaning that there were no no-treatment controls for the 6-week-delay experiments. Lastly is the notion of “lysis of bacteria from outside,” which I interpret as reference to “lysis from without” (Abedon, 2011) but which the authors appear to be equating, incorrectly, with the concept of inundation (Payne and Jansen, 2001). Inundation therapy generally is assumed to involve bacteria killing though not necessarily also phage replication nor even bacterial lysis.

A much more substantive concern is that these authors have, in essence, made their reported treatment look *too easy*. This is not a criticism of the results presented but instead stems from a much more general question of just what constitutes a chronic bacterial infection as typically treated clinically using phage therapy. Thus, does this rabbit osteomyelitis model, impressive as it appears, come close to actually modeling antibiotic-tolerant chronic bacterial infections?

The authors indicate that chronic osteomyelitis is defined by bone inflammation that has lasted for at least 6 weeks. By contrast, by the time phage treatment has begun in the clinic, chronic infections typically have persisted, often despite ongoing antibiotic treatment, for up to many years, e.g., a median of 43 months as reported by Miedzybrodzki et al. (2012). Differences therefore may exist between chronic osteomyelitis at 6 weeks and that which phage therapists may encounter in the clinic, and particularly so given that there have been delays in treatment until after more conventional approaches have failed.

Drawing on the Kishor et al. (2016) study as well as others presented in Table 1, I would like to suggest five criteria for judging experimental animal infections as models for phage therapy of chronic infections as encountered in the clinic. As the first criterion should be assumed as a given, I start the list at zero:

0. Substantial delays, e.g., multiple days, weeks, or longer, following bacterial challenge; minimally including demonstration of some degree of infection stabilization prior to the onset of phage treatment.
1. Equivalent degrees of site preparation as used clinically prior to the onset of treatment, e.g., debridement in the case of wound infections.

Generally these should be followed with at least two of the following:

2. Demonstration, in most cases, of a *lack* of adequate treatment success without multiple, temporally separated phage applications over relatively long periods, i.e., if multiple doses over weeks are required in the clinic to adequately combat chronic infections then success using only a single dose in the laboratory ought to be suspect.
3. Demonstration, in many cases, of development of biofilms prior to treatment.
4. Demonstration of physiological development, by model infections, of antibiotic tolerance.

The latter point in a sense is an operational definition since chronic infections can become eligible for phage therapy due to a failure to be effectively treated using antibiotics—failures which are not necessarily consequences of genetically acquired resistance and particularly as associated with biofilm formation. Thus, chronic infection models for phage therapy in at least some cases should be ones for which antibiotics, as normally employed, have lost their ability to clear otherwise laboratory-sensitive bacteria over the course of infection development.

In conclusion, with few quibbles, I want to stress that I am delighted with Kishor et al.'s efforts. But, as with good science generally, as many questions are raised by their study as have been answered.

## Author contributions

The author confirms being the sole contributor of this work and approved it for publication.

### Conflict of interest statement

The author declares that the research was conducted in the absence of any commercial or financial relationships that could be construed as a potential conflict of interest.
